# Prevalence and factors associated with anemia among patients with type 2 diabetes mellitus in northeastern Thailand: A cross-sectional study

**DOI:** 10.1371/journal.pone.0358120

**Published:** 2026-09-15

**Authors:** Khomsan Yooyen, Seubsiri Bunditpirom, Supan Fucharoen, Attawut Chaibunruang, Hataichanok Srivorakun, Supawadee Yamsri

**Affiliations:** 1 Doctor of Philosophy in Medical Sciences Program, Graduate School, Khon Kaen University, Khon Kaen, Thailand; 2 The Centre for Research and Development of Medical Diagnostic Laboratories, Faculty of Associated Medical Sciences, Khon Kaen Universyugbity, Khon Kaen, Thailand; 3 Nonsaad Hospital, Udon Thani, Thailand; Ankara University: Ankara Universitesi, TÜRKIYE

## Abstract

**Introduction:**

Anemia is a common complication in individuals with type 2 diabetes mellitus (T2DM), particularly in regions where thalassemia and iron deficiency are prevalent. However, the relative contribution of genetic hemoglobin disorders in this context has not been well characterized. This study aimed to determine the prevalence of anemia and to identify factors associated with its occurrence among patients with T2DM in northeastern Thailand.

**Methods:**

A cross-sectional study was conducted among 210 patients with T2DM attending two community health-promoting hospitals in northeastern Thailand. Data on sociodemographic characteristics, hematologic indices, renal function, iron status, and thalassemia genotypes were collected. Anemia was defined according to World Health Organization criteria. Factors associated with anemia were evaluated using multivariable logistic regression analysis, and adjusted odds ratios (AORs) with 95% confidence intervals (CIs) were reported.

**Results:**

The prevalence of anemia was 54.3%. Thalassemia and related hemoglobinopathies were present in 60.5% of participants, and iron deficiency in 39.5%. Independent predictors of anemia included two or more α-globin gene defects or Hb E co-inherited with α-thalassemia (adjusted OR: 37.8; p = 0.002), Hb E trait (OR: 3.6; p = 0.006), iron deficiency (OR: 3.7; p < 0.001), and chronic kidney disease stages 2 (OR: 3.5; p = 0.002) and 3–4 (OR: 15.7; p < 0.001).

**Conclusion:**

Anemia was highly prevalent among patients with T2DM in this community-based cohort from northeastern Thailand. Thalassemia-related genotypes, iron deficiency, and chronic kidney disease were significantly associated with anemia. These findings highlight the importance of considering inherited hemoglobin disorders when evaluating anemia in T2DM patients in hemoglobinopathy-endemic regions.

## Introduction

Diabetes Mellitus (DM) is a global chronic epidemic characterized by impaired glycemic control due to defects in insulin secretion and action [1–3]. According to the World Health Organization (WHO), approximately 422 million people worldwide are affected by diabetes, with around 1.5 million deaths directly attributed to the disease each year [4]. T2DM accounts for approximately 90% of all diabetes cases. It is primarily caused by chronic hyperinsulinemia, which leads to decreased insulin secretion, insulin resistance, or both [5,6]. Thailand is among the countries in Asia with a high prevalence of diabetes. According to the International Diabetes Federation (IDF) in 2021, the prevalence of diabetes in adults in Thailand was 10.5%. This high prevalence contributes to an increasing number of diabetes-related deaths and complications, posing a significant public health challenge. The high prevalence of T2DM is related to the development of chronic diabetes complications, it presents significant problems such as endothelial and vascular disease, diabetic neuropathy, diabetic retinopathy, and chronic kidney disease (CKD) [7,8]. Moreover, studies have shown that the prevalence of anemia is higher in patients with T2DM compared to non-diabetic individuals [9,10].

Anemia is a condition characterized by a reduction in the number of red blood cells (RBCs) or hemoglobin (Hb) concentration in the blood, leading to decreased oxygen transport capacity. Anemia can result from various factors including nutritional deficiencies (iron, folate, vitamin B12), chronic diseases, and genetic disorders [11–13]. Anemia is a common feature in T2DM. The prevalence of anemia in T2DM patients ranges from 14–49% [13–16]. This variation can be attributed to several factors such as difference ethnicities, demographic characteristics and diabetic complications. Among diabetic complications, CKD is a major complication that leads to reduced production of erythropoietin, a hormone that stimulates red blood cell production, thus contributing to anemia in T2DM patients [17]. The etiology of anemia in T2DM is multifactorial, involving nutritional, genetic, and renal factors. Of these, genetic disorders such as thalassemia and hemoglobinopathies as well as iron deficiency can play significant roles. The burden of these risk factors is particularly challenging in areas with a high prevalence of thalassemia and iron deficiency.

It has been known that northeast Thailand has a higher prevalence of thalassemia as compared to other regions, particularly hemoglobin E (Hb E). Thalassemia has been reported as a primary contributor to anemia in affected populations, whereas iron deficiency has also been identified as an additional significant factor contributing for developing anemia [18–21]. However, study on risk factors of anemia in context of thalassemia and iron deficiency among diabetic patients is limited. To address this knowledge gap, this study aims to evaluate the prevalence of anemia in northeastern Thai patients with T2DM and investigate its relationship to thalassemia genes and iron deficiency.

## Materials and methods

### Study design and subjects

This cross-sectional study was conducted between April and May 2023 at two community health-promoting hospitals in Nong-Saeng district, Udon Thani province, northeastern Thailand. Patients with registered T2DM in these hospitals routinely attend annual follow-up visits for diabetes management and laboratory monitoring. The present study was conducted during this period, and patients attending their scheduled annual check-up were invited to participate consecutively. Therefore, the study period reflects the recruitment window rather than the duration of clinical follow-up.

The study was approved by the Ethics Committee of Khon Kaen University (HE662002). All methods were carried out in accordance with relevant guidelines and regulations. Voluntary informed consent was obtained from all participants, and the confidentiality of the information provided was guaranteed. Access to participants’ information was restricted to only the principal investigator and the project administrator. Therefore, there are 289 T2DM patients registered in these hospitals. A sample size was calculated using a formula for infinite population proportion. With an expected anemia prevalence of 49% and 95% confidence interval at 10% marginal error, the minimum number of participants required for the study was 166. However, to increase statistical power and account for missing data, all participants who provided informed consent were recruited for the study. Therefore, on a voluntary basis, a total of 210 T2DM patients were enrolled.

Before specimen and data collection, the healthcare providers at the two community health-promoting hospitals announced the research project to the T2DM patients. The project staff explained the aims of the research, research procedures, benefits, and the risks of participating in the research to all patients. The patients made a voluntary decision to or not to participate, and each participant was asked to sign a consent form. Socio-demographic information, including age, sex, marital status, education, occupation, and personal income, was obtained through interviews using a questionnaire and recorded by healthcare staff. Participants had the freedom to decline any question or withdraw from the study at any time. Medication treatment history and patient complications were obtained from patients’ medical records from each community health-promoting hospital database. On the same day as the participants’ annual health check-up, an additional 5 milliliters (ml) of venous blood were drawn from each participant, with 2 ml collected in K3-EDTA tube and 3 ml collected in clotting tube. All blood samples were stored in a cool box and transported to a hospital for complete blood count (CBC) analysis and serum separation. The left-over EDTA blood and serum samples were stored at 2–8 °C and transported to the Thalassemia Service Unit (TSU), the Centre for Research and Development of Medical Diagnostic Laboratories (CMDL) at Khon Kaen University, Thailand, for further laboratory investigations of thalassemia and hemoglobinopathies. Laboratory data including fasting blood sugar, creatinine, total cholesterol, triglycerides, low-density lipoprotein (LDL), high-density lipoprotein (HDL), microalbumin, urine protein, uric acid, eGFR, and HbA1C, were obtained from the participants’ annual health check-up.

### Laboratory investigations

Complete blood counts were determined using automated analyzers (Sysmex XN-550, Sysmex Co, Kobe, Japan.) within 4 hours after blood collection. Iron profiles, including serum ferritin, serum iron, transferrin saturation, and total iron-binding capacity (TIBC), were analyzed in serum using automated analyzers (Beckman Coulter UniCel DxI 800, Beckman Coulter Inc., CA, USA). Ferritin was measured using a two-site immuno-enzymatic assay. Serum iron and transferrin were measured using an Fe reagent via a timed endpoint method and transferrin reagent via the turbidimetric method, respectively. Transferrin saturation and TIBC values were calculated. Additionally, high-sensitivity C-reactive protein (hsCRP) was measured in serum using the near-infrared particle immunoassay rate method with the same automated analyzers.

For investigation of thalassemia and hemoglobinopathies, hemoglobin analysis was performed using capillary zone electrophoresis (Capillarys II; Sebia, Leisse, France). Thalassemia and hemoglobinopathies were diagnosed according to standard guidelines. Diagnosis of β-thalassemia was made in individuals with normal hemoglobin pattern and Hb A2 > 3.5% [22]. β-thalassemia mutations were identified using an allele-specific PCR (ASPCR) approach [23]. α^0^-thalassemia genes, including SEA and THAI deletion, were investigated in suspected cases while α^+^-thalassemia genes, including 3.7 kb deletion, 4.2 kb deletion, Hb Constant Spring and Hb Paksé, were investigated in all subjects using multiplex gap-PCR and allele specific PCR (ASPCR), as described previously [24–26].

Anemia was defined as Hb < 13.0 g/dL in males and < 12.0 g/dL in females according to the World Health Organization (WHO). Severity of anemia was further graded as mild (Hb = 11.0–12.9 g/dL for males and 11.0–11.9 g/dL for females), moderate (Hb 8.0–10.9 g/dL), and severe (Hb < 8.0 g/dL) [27]. CKD was diagnosed by clinician using the criteria; eGFR values: ≥ 90 ml/min/1.73 m^2^ for stage 1, 60–89 ml/min/1.73 m^2^ for stage 2, 30–59 ml/min/1.73 m^2^ for stage 3, and 15–29 ml/min/1.73 m^2^ for stage 4 [28].

Iron deficiency was defined as serum ferritin <70 µg/L or transferrin saturation <20%. The ferritin threshold of <70 µg/L was follows the WHO guideline on ferritin use for assessing iron status, which recommends a higher cut-off (<70 µg/L in adults) in individuals with infection, inflammation, or chronic disease because ferritin is an acute-phase reactant. Therefore, transferrin saturation was also considered to reduce misclassification. [29].

Dyslipidemia was identified by at least one abnormal lipid profile indicator which includes total cholesterol, LDL, HDL, and triglycerides [30]. Body mass index (BMI) was categorized according to WHO criteria for Asian populations as follows: underweight (< 18.5 kg/m^2^), normal weight (18.5–24.9 kg/m^2^), overweight (25.0–29.9 kg/m^2^), and obesity (≥ 30 kg/m^2^) [31].

### Statistical analysis

Descriptive statistics were used to summarize the characteristics of the study population. All variables were categorized and presented as frequencies and percentages. Percentages and 95% confidence intervals (95% CI) were calculated to estimate the prevalence of anemia, iron deficiency (ID), and thalassemia among the participants. To identify factors associated with anemia, potential variables were initially assessed using the Chi-squared test. These variables included age group, sex, body mass index (BMI), fasting blood sugar (FBS), glycated hemoglobin (HbA1C), dyslipidemia, chronic kidney disease (CKD) stages, hypertension, use of antihypertensive drugs, use of antihyperlipidemic drugs, use of aspirin, iron deficiency, and thalassemia genotypes. Variables with p-value < 0.25 were selected for inclusion in a multivariable logistic regression model. Adjusted odds ratios (AORs) with 95% confidence intervals (CI) were calculated to determine which factors were independently associated with anemia. A p-value < 0.05 was considered statistically significant. All statistical analyses were conducted using Stata software version 18 (Stata Corp, Texas, USA).

## Results

### General characteristics of study participants

A total of 210 patients with T2DM participated in this community-based study. The majority were female (70.5%) and aged between 60–69 years (40.5%), followed by those aged <60 years (35.7%) and ≥70 years (23.8%). Most participants were married (69.0%) and had attained junior high school education or lower (92.9%). Over half were business owners (52.8%), and 75.2% reported monthly incomes of less than 5,000 Baht. The distribution of sociodemographic and clinical characteristics is summarized in Table 1.

**Table 1:** General characteristics and laboratory findings of patients with T2DM (n = 210).

Variables	Categories	n	Frequency (%)
Age group	< 60 years	75	35.7
	≥ 60 years	135	64.3
Sex	Female	148	70.5
	Male	62	29.5
Marital status	Married	145	69.0
	Separated	38	18.1
	Widowed	18	8.6
	Single	9	4.3
Educations	Junior high school or lower	195	92.9
	Senior high school	15	7.1
Occupations	Business owner	111	52.8
	Farmer	9	4.3
	Unemployed	90	42.9
Personal incomes (Baht/month)	< 5,000	158	75.2
	5,000-10,000	27	12.9
	> 10,000	12	5.7
	Undisclosed	13	6.2
BMI^*^	Underweight	15	7.1
	Normal	98	46.7
	Overweight	68	32.4
	Obesity	29	13.8
FBS	<126 mg/dL	74	35.2
	≥ 126 mg/dL	136	64.8
HbA1C	≤ 7.0%	92	43.8
	> 7%	118	56.2
Dyslipidemia^**^	No	28	13.3
	Yes	182	86.7
CKD stages^***^	Stage 1	72	34.3
	Stage 2	93	44.3
	Stage 3	44	20.9
	Stage 4	1	0.5
Hypertension	Yes	140	66.7
	No	70	33.3
Smoking	Yes	9	4.3
	No	201	95.7
Alcohol drinking	Yes	18	8.6
	No	192	91.4
Use of antihypertensive drug	Yes	138	65.7
	No	72	34.3
Use of antihyperlipidemic drug	Yes	92	43.8
	No	118	56.2
Use of Aspirin	Yes	92	43.8
	No	118	56.2

*Categorized according to WHO criteria for Asian populations: underweight (<18.5 kg/m^2^), normal weight (18.5–24.9 kg/m^2^), overweight (25.0–29.9 kg/m^2^), and obesity (≥30 kg/m^2^).

**Defined as having at least one abnormal lipid profile indicator.

***Chronic kidney disease staging based on eGFR: stage 1 (≥90 mL/min/1.73 m^2^), stage 2 (60–89 mL/min/1.73 m^2^), stage 3 (30–59 mL/min/1.73 m^2^), and stage 4 (15–29 mL/min/1.73 m^2^).

Regarding metabolic and clinical parameters, 46.7% of participants had a normal body mass index (BMI), while 32.4% were overweight, 13.8% were classified as obese, and 7.1% were underweight. Elevated fasting blood sugar (FBS ≥ 126 mg/dL) was observed in 64.8% of participants, and 56.2% had poor glycemic control with HbA1C levels >7%. Dyslipidemia was prevalent in 86.7% of the cohort, and 66.7% had a diagnosis of hypertension. In terms of renal function, 44.3% and 20.9% of participants were classified as having chronic kidney disease (CKD) stages 2 and 3, respectively. The use of antihypertensive drugs was reported in 65.7% of the study population.

### Prevalence of anemia, thalassemia, and iron deficiency

Anemia was identified in 114 out of 210 participants, corresponding to a prevalence of 54.3% (95% CI = 47.5–61.0) (Table 2). Mild anemia was the most common form, observed in 32.4% of participants. Moderate anemia was found in 20.5%, while severe anemia was uncommon (1.4%). Thalassemia and other hemoglobinopathies were identified in 127 participants, representing 60.5% (95% CI = 53.9–67.6). Iron deficiency was found in 83 participants (39.5%, 95% CI = 33.5–46.7), based on serum ferritin and transferrin saturation levels.

**Table 2:** Prevalence of anemia, thalassemia, and iron deficiency among patients with T2DM (n = 210).

Conditions	n	Frequency (%)	95%CI
**Anemia** ^*^	114	54.3	47.5 - 61.0
*Mild*	*68*	*59.6*	*50.6 - 68.7*
*Moderate*	*43*	*37.7*	*28.8 - 46.6*
*Severe*	*3*	*2.6*	*0.0 - 5.6*
**Thalassemia**	127	60.5	53.9 - 67.1
**ID** ^**^	83	39.5	32.9 - 46.1

*Anemia was defined as hemoglobin < 13.0 g/dL for males and < 12.0 g/dL for females. Severity was further classified as follows: mild anemia (Hb 110–129 g/L for males, 110–119 g/L for females), moderate anemia (Hb 80–109 g/L), and severe anemia (Hb < 80 g/L).

**Iron deficiency (ID) was defined as serum ferritin < 70 µg/L or transferrin saturation < 20%.

### Genotypic distribution of thalassemia and hemoglobinopathies

A total of 16 different genotypes of thalassemia and hemoglobinopathies were identified among the 210 participants, as summarized in Table 3. Of these, 127 individuals (60.5%) carried at least one abnormal genotype. The most frequently observed genotypes were heterozygous Hb E (21.4%) and heterozygous α⁺-thalassemia (13.3%). Other thalassemic Hb variants included heterozygous Hb Constant Spring (3.8%), homozygous Hb E (2.9%), homozygous α⁺-thalassemia (1.0%), and heterozygous α^0^-thalassemia (1.0%). Only one case of heterozygous β-thalassemia was encountered (0.5%). Regarding complex genotypes, as much as 11.0% of participants carried compound heterozygous Hb E with α⁺-thalassemia. Additional complex genotypes included Hb E with homozygous α⁺ -thalassemia (0.5%), Hb E with Hb Constant Spring (1.0%), and compound α^0^-/α⁺-thalassemia (1.0%). Rare genotypes such as Hb E co-inherited with compound α⁺-thalassemia/Hb Pakse’ and Hb E with α^0^-thalassemia were observed in each one case.

**Table 3:** Thalassemia genotypes among 210 patients with T2DM (n = 210).

Thalassemia genotypes	n	Frequency (%)
**α-β-thalassemia**		
Heterozygous α^+^-thalassemia	28	13.3
Heterozygous α^0^-thalassemia	2	1.0
Homozygous α^+^-thalassemia	2	1.0
Heterozygous β^0^-thalassemia	1	0.5
**Thalassemic Hb variants**		
Heterozygous Hb E	45	21.4
Homozygous Hb E	6	2.9
Heterozygous Hb Constant Spring	8	3.8
**Complex interaction**		
Heterozygous Hb E with heterozygous α^+^-thalassemia	23	11.0
Heterozygous Hb E with Hb Constant Spring	2	1.0
Homozygous Hb E with heterozygous α^+^-thalassemia	2	1.0
Heterozygous Hb E with heterozygous α^0^-thalassemia	1	0.5
Heterozygous Hb E with homozygous α^+^-thalassemia	1	0.5
Heterozygous Hb E with compound heterozygous α^+^-thalassemia/ Hb Pakse’	1	0.5
Homozygous Hb E with homozygous α^+^-thalassemia	1	0.5
Compound heterozygous α^0^-thalassemia/α^+^-thalassemia	2	1.0
Compound heterozygous α^+^-thalassemia/ Hb Constant Spring	2	1.0
**Total**	**127**	**60.5**

### Factors associated with anemia

Preliminary analysis was conducted to identify potential factors associated with anemia among patients with T2DM. Based on Chi-squared test results (p-value < 0.25), seven candidate variables including thalassemia, iron deficiency (ID), chronic kidney disease (CKD) stages, age, body mass index (BMI), fasting blood sugar (FBS), and the use of aspirin, were subsequently included in the multivariable analysis to assess their independent associations with anemia.

Multivariable logistic regression analysis was conducted to identify significant predictors of anemia among T2DM patients. The results are presented in Table 4. Among the thalassemia-related genotypes, two groups were significantly associated with anemia. Participants carrying two or more α-globin gene deletions or compound genotypes, including Hb E co-inherited with α⁺- or α^0^-thalassemia, showed the strongest association with anemia, although the confidence interval was wide because of the small number of participants in this subgroup (adjusted OR = 37.8; 95% CI: 4.0–360.2; *P* = 0.002). In addition, participants with Hb E trait alone were also significantly associated with anemia (adjusted OR = 3.6; 95% CI: 1.4–9.2; *P* = 0.006). In contrast, all participants with homozygous Hb E had anemia, odds ratios for this group could not be estimated using conventional logistic regression due to complete separation.

**Table 4:** Multivariable logistic regression analysis of factors associated with anemia among patients with T2DM.

Factors (n)	Anemia n (%)	Crude OR	Adjusted OR	95% CI	P-value
**Thalassemia**					
Normal (n = 83)	36 (43.4)	1 (Ref)	1 (Ref)		
Single α-gene defect (n = 36)	16 (44.4)	1.0	1.5	0.6-3.7	0.442
Two (or more) α-gene defects* (n = 12)	11 (91.7)	14.4	37.8	4.0-360.2	0.002
Hb E trait (n = 45)	28 (62.2)	2.2	3.6	1.4-9.2	0.006
Hb E trait with one α-gene defect (n = 25)	14 (56.0)	1.7	2.0	0.7-5.8	0.204
Homozygous Hb E** (n = 9)	9 (100.0)	NA	NA	NA	NA
**ID**					
Non-ID (n = 127)	55 (43.3)	1 (Ref)	1 (Ref)		
ID (n = 83)	59 (71.1)	3.2	3.7	1.8-7.7	< 0.001
**CKD stages**					
Stage 1 (n = 72)	21 (29.2)	1 (Ref)	1 (Ref)		
Stage 2 (n = 93)	54 (58.1)	3.4	3.5	1.6-7.7	0.002
Stage 3 and stage 4 (n = 45)	39 (86.7)	15.8	15.7	5.0-49.9	< 0.001
**Age**					
< 60 (n = 75)	21 (33.9)	1 (Ref)	1 (Ref)		
≥ 60 (n = 135)	93 (62.8)	3.3	2.4	1.1-5.3	0.029

OR, odds ratio; AOR, adjusted odds ratio; CI, confidence interval; ID, iron deficiency; CKD, chronic kidney disease.

*Includes cases with Hb E co-inherited with two α-gene defect.^**^Includes individuals with homozygous Hb E co-inherited with α-thalassemia.^NA^ Indicates not applicable because all individuals in this category had anemia, resulting in complete separation.

**Fig 1:**
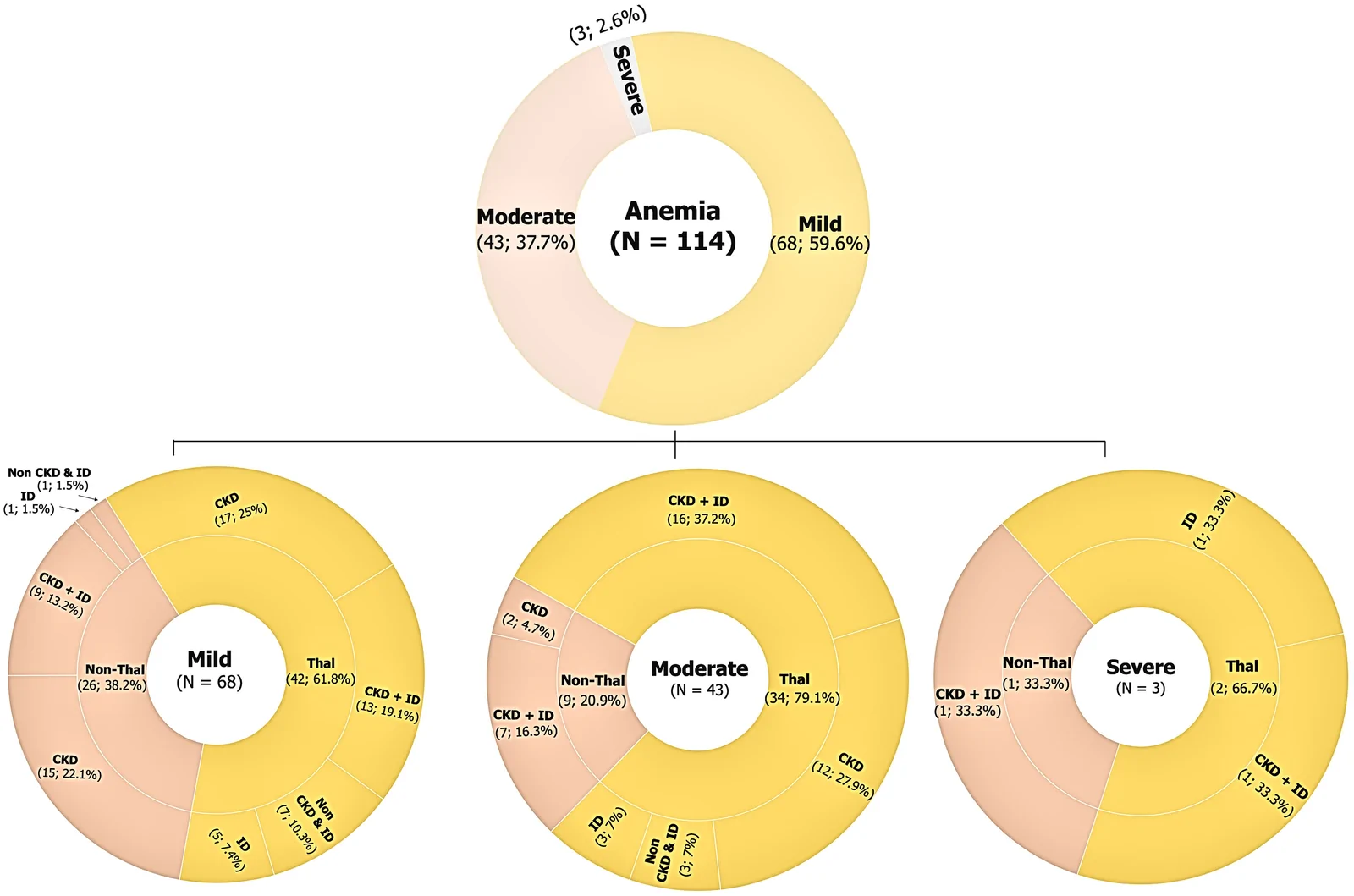
Sunburst chart of factors associated with anemia among anemic patients with T2DM (n = 114). Anemia cases were categorized into mild, moderate, and severe. The inner ring separates anemic patients according to the presence or absence of thalassemia. The outer ring depicts additional factors associated with anemia identified from the multivariable logistic regression model, including iron deficiency and chronic kidney disease (CKD) stages 2–4.

In addition to thalassemia, iron deficiency was strongly associated with anemia (adjusted OR = 3.7; 95% CI: 1.8–7.7; p < 0.001). Renal impairment demonstrated a dose-response relationship: patients with CKD stage 2 had elevated odds of anemia (adjusted OR = 3.5; 95% CI: 1.6–7.7; *P* = 0.002), while those with CKD stages 3–4 exhibited markedly higher risk (adjusted OR = 15.7; 95% CI: 5.0–49.9; *P* < 0.001). The contribution of these factors is visualized in Fig 1, which summarizes the distribution of etiologies among the 114 anemic patients.

## Discussion

This study evaluated the prevalence of anemia and its associated factors among patients with T2DM in a community-based setting in northeastern Thailand. Our findings showed that anemia was common, affecting more than half of the participants (54.3%), with mild anemia being the most frequent form. This prevalence is higher than that reported in previous hospital- and community-based studies in Thailand and other countries [5–8,32]. In settings where thalassemia is uncommon, the prevalence of anemia among T2DM patients has generally been reported to range between 20–35% [33,34]. These comparisons emphasize that the markedly higher prevalence of anemia observed in our cohort cannot be explained by acquired factors alone, but likely reflects the additional burden of thalassemia in northeastern Thailand, where hemoglobinopathies are endemic.

According to our findings, thalassemia and hemoglobinopathies were detected in 60.5% of participants, the data indicating a high prevalence of thalassemia and related hemoglobinopathies among individuals with T2DM in this community-based cohort. This finding is consistent with previous population-based studies conducted in northeastern Thailand, where carrier rates between 51% and 60.7% have been reported in both general adult populations and antenatal cohorts [18,21,35]. Similarly, a high prevalence of 67.7% was also observed among residents of communities near the Thailand–Lao PDR–Cambodia border [36]. Furthermore, ID was identified in 39.5% of participants, indicating a common condition among individuals with T2DM in a community setting. This prevalence is notably higher than that reported in the general population across several studies in northeastern Thailand. As reported in previous studies, ID was reported in only 6.2% of pregnant women in northeastern Thailand [18], 23.2% of women of reproductive age in the same region [21], and 10.5% of the general population in the Thailand–Lao PDR–Cambodia border area [36]. Given this high overall frequency, therefore, we further characterized the spectrum of thalassemia and hemoglobinopathy genotypes observed in this cohort.

To identify potential factors associated with anemia in this population, a preliminary analysis using the Chi-squared test was conducted. Seven candidate variables including thalassemia, iron deficiency (ID), chronic kidney disease (CKD) stages, age, body mass index (BMI), fasting blood sugar (FBS), and the use of aspirin, met the selection criteria (p < 0.25) and were included in the multivariable logistic regression model. The analysis identified several factors that were independently associated with anemia among patients with T2DM. Notably, the strongest association was observed in individuals carrying two or more α-globin gene deletions, including compound genotypes such as Hb E co-inherited with α⁺- or α^0^-thalassemia (AOR = 37.8; 95% CI: 4.0–360.2). However, this finding should be interpreted with caution because of the small number of participants in this subgroup, as reflected by the wide confidence interval around the effect estimate. Even carriers of Hb E trait alone were found to have a significantly elevated risk of anemia.

In regions where inherited hemoglobin disorders are highly prevalent, such as northeastern Thailand, thalassemia has consistently been identified as a major contributor to anemia. Our findings align unexpectedly with evidence from community-based studies in diverse populations, including adolescents, the elderly, women of reproductive age, and pregnant women [18,20,21,37–39]. These studies demonstrate that thalassemia and hemoglobinopathies are often more common than iron deficiency as the leading cause of anemia, particularly in endemic areas. In northeastern Thailand, the carrier rate of thalassemia can exceed 50%, with Hb E trait and α⁺-thalassemia being the most commonly observed genotypes [20,21,37]. Although Hb E trait is typically regarded as clinically mild, increasing evidence suggests that it may not be entirely benign in the presence of chronic diseases. In T2DM, chronic inflammation, oxidative stress, and metabolic dysregulation may impair erythropoiesis and shorten red blood cell survival, potentially exacerbating anemia in individuals with underlying hemoglobin disorders [40,41].

Of note, all individuals with homozygous Hb E (Hb EE) in this study had anemia. Because of complete separation, odds ratios for this group could not be estimated using conventional logistic regression. While this observation is consistent with previous reports indicating increased anemia risk in Hb EE individuals [18], the small number of cases in this subgroup limits the ability to draw definitive conclusions. Taken together, thalassemia, particularly in compound heterozygous and homozygous forms, were strongly associated with anemia in this cross-sectional cohort; however, causality cannot be inferred.

Iron deficiency (ID) was also independently associated with anemia in this cohort (adjusted OR = 3.7). Approximately 39.5% of participants met the criteria for iron deficiency, indicating that nutritional factors remain an important contributor to anemia even in regions with a high prevalence of hemoglobinopathies. In this study, iron deficiency was defined using a ferritin threshold of <70 µg/L or transferrin saturation <20%, in accordance with WHO recommendations for individuals with chronic inflammatory conditions. Because ferritin is an acute-phase reactant and may be elevated in T2DM, this higher threshold was considered appropriate. Comparative data from non-endemic settings show variable prevalence; for example, a cross-sectional study in India reported a higher ID prevalence of 63.5% in T2DM patients [11]. These discrepancies emphasize that nutritional causes of anemia in T2DM can be particularly important in regions with high genetic predisposition to anemia. Moreover, the pathophysiology of ID in T2DM may be multifactorial, involving poor dietary intake, menstrual losses, gastrointestinal bleeding, and diabetes-related GI complications such as gastritis or malabsorption, especially among long-term metformin users or patients with CKD [13,42–44]. Supporting this, community-based studies from northeastern Thailand have demonstrated high ID prevalence in women of reproductive age and pregnant women, ranging from 6.2% to 23.2%, [18,20,21] whereas our cohort exhibits a markedly higher prevalence. Recent evidence also demonstrates that vitamin B12 deficiency is common in metformin-treated patients. For example, a study from Vietnam found that 22.1% of individuals with T2DM on metformin had B12 deficiency, especially those with a median treatment duration of 10 years and a daily dose of 1700 mg [43]. Together with ID, these findings highlight the role of nutritional factors as important contributors to anemia in diabetes.

Interestingly, although more than half of the participants (56.2%) had poor glycemic control, HbA1C was not independently associated with anemia in the multivariable model. Although chronic hyperglycemia is known to contribute to microvascular damage, chronic inflammation, and oxidative stress, which may impair erythropoiesis or shorten erythrocyte lifespan [13,33,44], our findings suggest that poor glycemic control alone does not fully explain the high prevalence of anemia observed in this community. This suggests that, in populations with a high burden of inherited hemoglobin disorders and iron deficiency, these factors may have a stronger association with hemoglobin levels than glycemic status alone [12]. Nevertheless, poor glycemic control may indirectly contribute to anemia through mechanisms such as renal impairment and chronic inflammation [42].

In addition, renal impairment emerged as another major contributor to anemia in this cohort, chronic kidney disease (CKD) was found to be an independent and strong predictor of anemia among patients with T2DM. The association exhibited a dose-response relationship across CKD stages: participants with stage 2 CKD had an increased risk of anemia (adjusted OR: 3.5), while those with stages 3–4 demonstrated a markedly higher risk (adjusted OR: 15.7). This pattern aligns with well-established pathophysiological mechanisms in diabetic nephropathy, where declining renal function leads to impaired erythropoietin (EPO) production and contributes to anemia of chronic disease [45]. Several studies have shown that anemia is more prevalent and occurs earlier in diabetic patients with CKD than in those with CKD from other causes. Erythropoietin deficiency, iron dysregulation, and low-grade inflammation are key contributors to this condition. In particular, reduced EPO synthesis in the diseased kidney and elevated hepcidin levels in the context of chronic inflammation inhibit both iron mobilization and erythropoiesis, thereby compounding the risk of anemia [13,46]. The findings from this community-based study support these mechanisms and highlight the clinical relevance of CKD staging in predicting anemia severity. Previous studies suggest that even mild to moderate reductions in glomerular filtration rate (GFR) are associated with subclinical reductions in hemoglobin levels, which may go undetected unless actively monitored.

A key consideration in interpreting these findings is the complex and multifactorial nature of anemia in this population. In regions such as northeastern Thailand, where thalassemia is highly prevalent, baseline hemoglobin levels may already be influenced by genetic factors. Our findings suggest that anemia in T2DM patients likely reflects the combined effects of inherited hemoglobin disorders, nutritional deficiencies, and diabetes-related complications, rather than a single underlying cause.

Several limitations should be acknowledged. First, the cross-sectional design limits the ability to determine whether the identified factors actually cause anemia. Second, the study was conducted in two community hospitals within a single district, the findings may not be generalizable to all patients with T2DM in northeastern Thailand or to populations with different genetic, nutritional, or healthcare backgrounds. Third, some genotype categories contained small numbers of participants, leading to wide confidence intervals and reduced precision of the estimated associations. Fourth, although key causes of anemia including thalassemia, iron deficiency, renal function, and inflammation, were thoroughly assessed, other less common contributors such as vitamin B12 or folate deficiency were not evaluated. In addition, data on metformin use and vitamin B12 levels were not collected. Therefore, the potential contribution of long-term metformin use and associated vitamin B12 deficiency to anemia could not be assessed. Nevertheless, this study provides important insights into the multiple and interacting causes of anemia in patients with T2DM, particularly in regions with a high prevalence of thalassemia.

In conclusion, anemia was common among patients with T2DM in this community-based cohort from northeastern Thailand. Thalassemia-related genotypes, iron deficiency, and chronic kidney disease were independently associated with anemia. These findings support the consideration of inherited hemoglobin disorders, together with nutritional and renal factors, when evaluating anemia in T2DM patients in regions where hemoglobinopathies are endemic.

## Supporting information

S1 Data Raw data used in Tables 1–4 and Fig 1. (XLSX)
